# The Role of Saliva and Mouthwashes in the Detection and Reduction of Oral Viral Load: A Scoping Review

**DOI:** 10.3390/v17111509

**Published:** 2025-11-17

**Authors:** Flavia Vitiello, Romain Lan, Giovanna Orsini, Denis Bourgeois, Florence Carrouel

**Affiliations:** 1 Department of Clinical Sciences and Stomatology (DISCO), Università Politecnica delle Marche, 60126 Ancona, Italy; f.vitiello@pm.univpm.it (F.V.); g.orsini@staff.univpm.it (G.O.); 2 Laboratory “Health, Systemic, Process” (P2S), UR4129, University Claude Bernard Lyon 1, University of Lyon, 69008 Lyon, France; romain.lan@univ-lyon1.fr (R.L.); denis.bourgeois@univ-lyon1.fr (D.B.); 3 Laboratory Anthropologie Bio-Culturelle, Droit, Ethique et Santé (ADES), Aix-Marseille University, Centre National de la Recherche Scientifique (CNRS), Etablissement Français du Sang (EFS), 13005 Marseille, France

**Keywords:** oral cavity, oral viruses, saliva, SARS-CoV-2, viral load, mouthwash

## Abstract

**Background:** The oral cavity is an entry site and a reservoir for viruses. Viral particles accumulate in saliva, which serves as a diagnostic fluid and vehicle for transmission (droplets and aerosols). Antiseptic mouthwashes were proposed as adjunctive measures to temporarily reduce oral viral load. **Objectives:** This scoping review aims to investigate the role of the oral cavity in viral infections, focusing on saliva and the use of antiseptic mouthwashes to reduce salivary viral load. **Methods:** Following the PRISMA-ScR guidelines, PubMed, EMBASE, and Web of Science were searched for human studies (2015–2025) investigating oral viral infections, saliva, or mouthwashes. Eligible studies were classified and analyzed for population, intervention, and outcomes. **Results:** Twenty-three studies met inclusion criteria (sixteen randomized controlled trials and seven systematic reviews). All included studies focused exclusively on SARS-CoV-2, as no clinical evidence on other oral viruses met the eligibility criteria. Saliva was consistently identified as a reliable, non-invasive specimen reflecting disease dynamics and transmission potential. Mouthwashes containing povidone-iodine, cetylpyridinium chloride, chlorhexidine, hydrogen peroxide or β-cyclodextrin–citrox produced measurable but short-lived reductions in salivary viral load. Heterogeneity and lack of standardized outcomes limited comparability. **Conclusions:** Antiseptic mouthwashes can provide a transient and complementary reduction in salivary viral load, particularly before aerosol-generating procedures; however, they should be regarded only as adjunctive measures and not as substitutes for standard infection-control protocols.

## 1. Introduction

The oral microbiome is a complex ecosystem composed of approximately 1000 species of bacteria, fungi, viruses, archaea, and protozoa [1,2]. This community of microorganisms is highly dynamic and constantly interacts with its host and environment. While minor environmental disturbances are generally self-limiting and compensated by homeostatic mechanisms, major alterations can disrupt the ecological balance, leading to dysbiosis [1].

The oral cavity provides a portal of entry and a replication site for a wide range of viruses. Respiratory viruses such as influenza, respiratory syncytial virus, SARS-CoV-2, herpesviruses including herpes simplex virus, cytomegalovirus, human papillomavirus, all exhibit tropism for the oral mucosa [3]. The oral viral tropism leads to the accumulation of viral particles in saliva, making the oral environment a critical reservoir for detection, prognosis, and transmission [4].

Therefore, saliva can be utilized as a diagnostic fluid, offering a non-invasive and easily accessible means of viral detection, with the capacity to identify the stage of the disease and its infectious potential [5]. Moreover, saliva serves as a vehicle for transmission, with viral particles disseminated through salivary droplets and aerosols, particularly during verbal interactions, coughing or dental procedures, thereby facilitating person-to-person transmission [6]. Hence, preprocedural mouth rinsing has been widely used before routine dental clinical treatments to reduce the number of oral microorganisms and the risk of pathogen transmission [7].

Thus, infection control strategies have increasingly focused on interventions aimed at temporally reducing salivary viral load. Among these, antiseptic mouthwashes, such as povidone-iodine (PVP-I), cetylpyridinium chloride (CPC), chlorhexidine gluconate (CHX), and hydrogen peroxide (HP) have emerged as potential adjunctive measures to mitigate viral transmission risk [8]. While in vitro studies demonstrated broad virucidal properties of mouthwashes against enveloped viruses, including herpes simplex virus, influenza virus, and Middle East respiratory syndrome coronavirus [9,10], the clinical effectiveness and duration of action remain uncertain.

The aim of this scoping review is to map and synthesize the available evidence on viruses with oral viral tropism, their detection and monitoring through saliva, and the use of antiseptic mouthwashes as interventions investigated in relation to salivary viral load, with a particular focus on identifying knowledge gaps and informing future research perspectives.

## 2. Materials and Methods

The present scoping review followed the methodology outlined by the Preferred Reporting Items for Systematic reviews and Meta-Analyses extension for Scoping Reviews (PRISMA-ScR) [11] (Supplementary Table S1). The protocol was developed internally by the research team prior to data collection but was not publicly registered. This approach ensured methodological rigor, transparency, and reproducibility. We included studies of all designs, following established scoping review procedures. The process followed five key steps: (1) Identification of the research question and development of the search strategy; (2) Screening and selection of relevant studies; (3) classification of included studies; (4) data extraction and charting; (5) synthesis and reporting of the results.

### 2.1. Search Strategy and Study Selection

An electronic literature search was conducted by two examiners in the databases PubMed, EMBASE and Web of Science. The searches were performed on 30 July 2025 and included studies published from June 2015 to June 2025. The search strategies used both MeSH and free terms keywords, combined using Boolean operators (AND, OR) and were adapted for each database syntax.

The search terms were (Viruses[MeSH Terms] OR virus OR Herpesvirus OR Herpes simplex virus OR Epstein–Barr virus OR Cytomegalovirus OR Human papillomavirus OR SARS-CoV-2 OR coronavirus OR influenza virus OR respiratory viruses) AND (Mouth[MeSH Terms] OR Saliva[MeSH Terms] OR saliva OR oral OR mouth OR oral mucosa OR salivary gland OR tongue OR gingiva) AND (transmission OR clinical manifestation OR symptoms OR oral lesions OR diagnosis OR detection OR screening OR pathogenesis OR contamination) AND (Mouthwashes[MeSH Terms] OR mouthwash OR mouthwashes OR rinse OR oral rinse OR antiseptic OR antiviral). After merging the results from the three databases, duplicate records were removed.

In addition, the reference lists of relevant reviews and included studies were screened manually to identify further eligible records. Grey literature (e.g., preprints, theses, conference abstracts) was not systematically searched, as the review focused on peer-reviewed sources to ensure methodological consistency and data quality.

### 2.2. Research Question

The PICO question was developed to guide the targeted investigation of the potential role of mouthwashes: In individuals with viral infections affecting the oral cavity (Population), does the use of antiseptic or antiviral mouthwashes (Intervention), compared to no mouthwash or placebo (Comparison), lead to a reduction in salivary viral load or infectivity (Outcome)?

### 2.3. Screening and Eligibility Criteria

Articles were included if they were (i) published between 2015 and 2025; (ii) written in the English language; (iii) fully accessible without restriction; (iv) publications featuring human studies; (v) focused on viruses of the oral cavity and their relationship with the oral microbiota or saliva.

Articles were excluded if they were (i) Case reports, case series, editorials, commentaries, letters, posters, conference abstracts or (ii) studies not relevant to the research questions.

The eligibility of the articles was assessed through an initial screening of titles and abstracts. For studies that appeared to meet the inclusion criteria, the full text was obtained and reviewed.

The selection process was carried out independently by two investigators (F.V. and F.C.), based on predefined inclusion and exclusion criteria. Discrepancies in study selection were resolved by consensus, with the involvement of a third author (D.B.) when agreement could not be reached between the initial reviewers.

### 2.4. Classification and Data Extraction

From the included publications, studies were classified by level of evidence: non- experimental (i.e., observational studies: cross-sectional, case–control, cohort), experimental studies (i.e., randomized controlled trials (RCTs)) and secondary evidence: systematic reviews and meta-analyses.

Data extraction was performed by one investigator and checked by a second. For each included study, the main data were extracted: citation details, study design, population, objectives, intervention, duration and follow-up, results, and conclusions.

### 2.5. Quality Assessment

The quality of included studies was evaluated by the two reviewers (F.V. and F.C.) using the National Institutes of Health’s study quality assessment tools [12]. In case of disagreement, the 2 reviewers discussed until a consensus was reached.

## 3. Results

### 3.1. Selection of Publications Included

**Figure 1:**
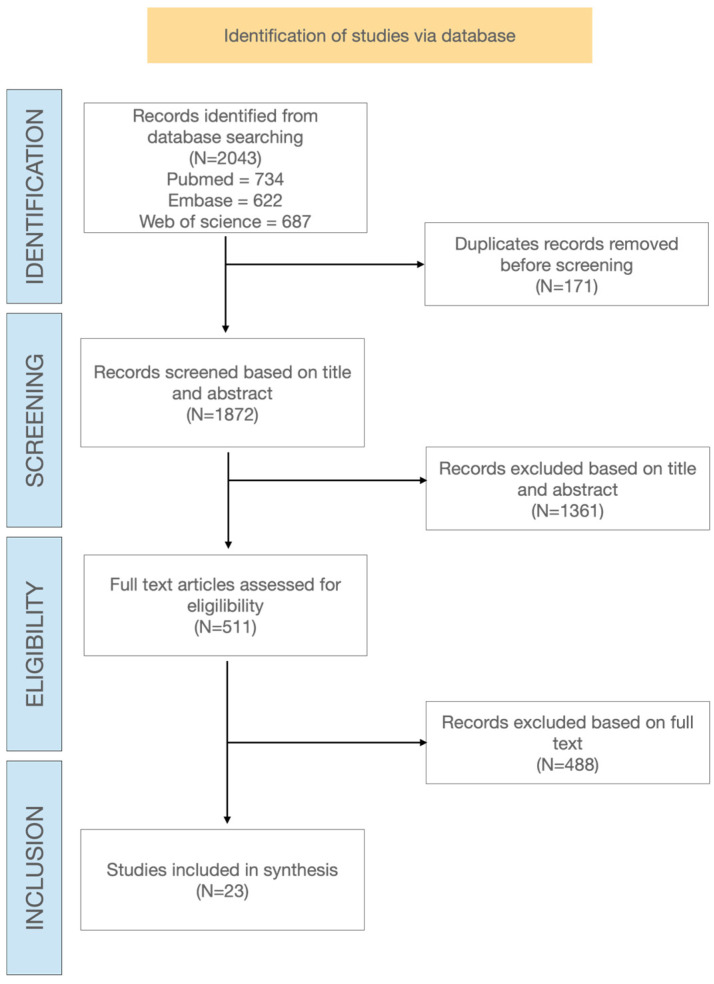
Flowchart of study and selection process.

Figure 1 shows the PRISMA-ScR study flowchart describing the different steps of article selection. From the initial database search, 2043 papers were identified. After removing duplicates, 1872 papers were screened at title and abstract level, and 511 potentially relevant full text articles were selected for eligibility assessment. Finally, 23 publications met the inclusion criteria and were included in the review.

Among the 23 included studies, there were 16 randomized controlled trials (RCTs) and 7 systematic reviews and meta-analyses.

### 3.2. Synthesis of the Results

#### 3.2.1. Saliva and Diagnosis

Three studies examined the diagnostic potential of saliva in the detection and monitoring of viral infections.

Among these, Verma et al. [13] demonstrated that salivary samples achieved sensitivity and specificity comparable to nasopharyngeal swabs. The study emphasized that saliva collection is non-invasive, easily repeatable, and well-suited for large-scale screening, thereby facilitating widespread surveillance and serial testing. These findings further support the role of saliva as a useful and reliable diagnostic specimen for respiratory viral infections.

Zhang et al. [14] reported that salivary viral RNA was consistently detectable in COVID-19 patients, and that changes in salivary viral concentration correlated with cycle threshold values obtained from molecular testing. Moreover, variations in viral load over time suggested that saliva could serve as a practical tool for monitoring disease dynamics.

Salivary viral load was also found to be closely associated with disease severity and clinical outcomes, in some cases even surpassing age as a predictor of mortality. Indeed, Espejo-Carrera et al. [15] reported that viral RNA was detectable in more than 90% of saliva samples collected from patients with confirmed COVID-19, including asymptomatic individuals. Moreover, higher salivary viral loads were significantly associated with greater disease severity and poorer clinical outcomes, suggesting a potential prognostic role. Viral concentrations reached up to 1.2 × 10^8^ copies/mL during the early stages of infection, reinforcing the concept that saliva functions both as a diagnostic specimen and a biomarker of disease progression.

Different saliva collection methods were reported across the included studies, including passive drooling [16,17,18,19] spitting [20,21,22], and self-collected cough-out saliva [23] which represents a mixture of saliva and respiratory secretions. Other studies employed oral or oropharyngeal swabs instead of pure saliva samples [24,25,26,27,28]. One study used a standardized saliva collection kit [29]. The variability in saliva sampling methods may influence viral load quantification, as passive drool primarily reflects glandular secretions, whereas spitting, cough-out, or swab-based approaches can include mucus and respiratory components, potentially contributing to differences in diagnostic sensitivity and viral load values reported across studies.

#### 3.2.2. Saliva and Transmission

Three studies focused on the role of saliva as a vehicle for viral transmission, especially in clinical and dental environments.

Koletsi et al. [30] investigated the role of saliva in viral transmission and demonstrated that it serves as both a reservoir and a source of viral dissemination. Salivary droplets and aerosols generated during clinical activities can carry infectious viral particles, posing a significant risk of airborne spread. This risk is particularly relevant in dental and medical settings, where aerosol-generating procedures (AGPs), such as ultrasonic scaling, orthodontic debonding, or restorative treatments, are routinely performed. The study showed that saliva acts as a major contributor to aerosol contamination, and that AGPs markedly amplify the dispersion of saliva-derived viral particles into the surrounding air, thereby elevating the risk of cross-infection for both healthcare professionals and patients.

García-Sánchez et al. [31] further investigated the contribution of saliva to aerosol contamination during dental procedures and confirmed that the oral cavity acts as a significant reservoir and vector for viral dissemination. Their results demonstrated that aerosols and splatters generated during AGPs frequently contain detectable viral RNA, indicating that saliva-derived particles play a critical role in the potential airborne spread of infection. This mechanism is particularly relevant in clinical contexts, where the close proximity between patients and healthcare professionals, combined with frequent aerosol generation, substantially increases the risk of cross-infection. The study highlights the need for enhanced protective measures and procedural modifications to minimize aerosol exposure during routine dental care.

Ebrahimi et al. [32] evaluated the effectiveness of pre-procedural interventions in mitigating the risk of viral transmission associated with salivary aerosols. Their findings showed that antiseptic mouth rinses administered prior to AGPs significantly reduced the microbial and viral load present in dental aerosols, indirectly confirming the pivotal role of saliva in transmission dynamics. Despite this, the authors emphasized that the current body of evidence remains predominantly focused on SARS-CoV-2, and that quantitative assessments of the infectious potential of saliva-derived aerosols are still limited. These results underscore the importance of integrating targeted infection control strategies into clinical practice and highlight the need for further research on aerosol-related transmission pathways.

#### 3.2.3. Mouthwashes

Sixteen RCTs [4,8,16,17,18,20,21,22,23,24,25,26,27,28,33,34] and one systematic review [35] investigated the effectiveness of antiseptic mouthwashes in reducing SARS-CoV-2 salivary viral load. A range of active agents was tested, including povidone-iodine (PVP-I), chlorhexidine (CHX), cetylpyridinium chloride (CPC), and hydrogen peroxide (H_2_O_2_), as well as less common formulations such as anionic phthalocyanine derivatives (APDs), benzalkonium chloride (BAC), and β-cyclodextrin–citrox mouthwash (CDCM). The selected studies reported that rinses were capable of achieving a measurable, albeit short-term, reduction in viral RNA concentrations, with varying levels of effectiveness across agents.

Several trials demonstrated that PVP-I was the most consistently effective agent, producing significant increases in Ct values and short-term reductions in salivary viral RNA compared with controls, although the effect was not sustained beyond one to two hours [17,18,23,26].

Perussolo et al. did not observe differences between CHX and the control groups, highlighting heterogeneity in clinical outcomes [21]. Onozuka et al. [34] observed that CPC achieved viral load reduction at 10 min post-rinse, though this effect was not maintained at 30–60 min, whereas in a subsequent trial [20] no significant differences were observed compared with placebo.

Ferrer et al. assessed H_2_O_2_, generally finding it ineffective in vivo despite promising in vitro results, with only minimal or inconsistent reductions reported [16]. Carrouel et al. evaluated CDCM in 176 COVID-19 outpatients and found a significant reduction in salivary SARS-CoV-2 load at 4 h post-rinse compared with placebo, with modest but greater reductions maintained at day 7 in patients with higher baseline viral loads [29]. Silva Santos et al. tested an APD formulation and found reductions in viral load together with improved clinical outcomes, including shorter hospital stay and no intensive care admissions [33].

Meister et al. confirmed the strong in vitro virucidal activity of BAC but reported only limited clinical benefits [28]. Two studies provided preliminary evidence of transient reductions in salivary viral load but were underpowered to detect significant effects [21,25].

Across the included trials, nearly all agents demonstrated some degree of salivary viral load reduction, although the effect was consistently transient (≤2 h), with only APD and CDCM showing sustained effects beyond this period. PVP-I consistently achieved the most reproducible reductions, whereas CHX and CPC showed variable outcomes, and H_2_O_2_ demonstrated limited efficacy despite strong in vitro activity.

Table 1 summarizes the characteristics and main findings of the 23 articles included in this review, providing an overview of the agents tested, study populations, outcomes, and duration of effect.

### 3.3. Synthesis of Results

Across the included studies, saliva consistently emerged as a reliable, non-invasive specimen for viral detection and as a potential vehicle for transmission. Most of the evidence was limited to SARS-CoV-2, with very few data addressing other respiratory or oral viruses. Antiseptic mouthwashes—including PVP-I, CPC, CHX, H_2_O_2_ and CDCM—produced measurable but short-lived reductions in salivary viral load, typically ≤ 2 h. Marked methodological heterogeneity, small sample sizes, and the absence of standardized endpoints prevented meta-analysis and limited comparability across studies. Together, these findings indicate that mouthwashes may offer temporary adjunctive benefits but do not provide sustained antiviral protection.

### 3.4. Quality of the Articles Included

Risk of bias by study design is presented in Table 2 and Table 3. Overall, the quality of the included studies was variable, ranging from low to high risk of bias. In systematic reviews and meta-analyses (Table 2), the high risk was mainly related to the criterion “bias assessed,” reflecting limited methodological transparency in some reports. In controlled intervention studies (Table 3), a moderate-to-high risk of bias was observed in several publications, particularly due to the lack of treatment allocation concealment, absence of blinding, small sample sizes, and incomplete adherence to the intervention.

## 4. Discussion

This scoping review provides a comprehensive synthesis of evidence on the role of saliva in viral diagnosis and transmission, and on the use of antiseptic mouthwashes as a potential adjunctive measure to reduce salivary viral load.

The findings highlighted that saliva functions both as a valuable diagnostic specimen and as a potential vehicle for viral dissemination, particularly in the context of the respiratory virus SARS-CoV-2.

All the studies included in this review focused exclusively on SARS-CoV-2. Since the onset of the pandemic, the role of saliva as a diagnostic and transmission medium has been a subject of extensive research [13,14,15], as has the potential of mouthwashes to reduce viral load in the oral cavity [23,31,35]. However, evidence on other respiratory or oral viruses, such as influenza, rhinovirus, herpesvirus, or coxsackievirus, remains almost completely absent, therefore the conclusions of this review should be interpreted within the context of SARS-CoV-2–related evidence. This limitation in the scope of research results in a restriction of the applicability of current findings and underscores a significant research gap. It is recommended that future studies expand the scope beyond SARS-CoV-2 in order to clarify whether the observed effects of antiseptic rinses and the diagnostic potential of saliva can be generalized to other pathogens.

A high degree of variability was observed across studies in terms of design, sample size, and experimental parameters. The concentration and exposure time of mouthwash formulations differed considerably, ranging from single-use rinses of 15 s to repeated applications lasting up to several minutes. Furthermore, the timing of post-rinse sampling and molecular quantification was inconsistent, with intervals varying from 10 min to 2 h.

In addition, differences in saliva sampling procedures, ranging from passive drooling and spitting to oral or oropharyngeal swabs, may have contributed to variability in viral load quantification across studies. Variations in sample composition, such as the presence of glandular secretions versus mucus or respiratory components, could affect the quantification of viral load and limit the comparability of findings across trials.

Moreover, the majority of studies employed molecular markers, such as the detection of viral RNA or cycle threshold alterations, as surrogate endpoints, without conducting a comprehensive assessment of viral infectivity or clinical transmission outcomes. However, the employment of randomized, double-blind, placebo-controlled designs is rare, which can result in potential bias and limited reproducibility [21,27].

Across all RCT included, the duration of antiviral effects was consistently short, generally lasting no more than two hours. Viral load reductions observed immediately after rinsing tended to return to baseline at subsequent measurements [17,23,26]. None of the studies assessed long-term viral suppression, sustained infectivity reduction, or clinical transmission prevention.

A significant limitation identified in the current literature is the lack of data on the safety of antiseptic mouthwashes and their effect on the oral microbiome. While agents such as PVP-I, CHX and CPC are generally considered safe for short-term use, few studies have included structured safety assessments or systematic monitoring of adverse events. Furthermore, none of the available trials evaluated the effects on oral microbiota, mucosal integrity or local immune responses. This restricts the interpretation of current findings, as reductions in viral load cannot be considered alongside potential adverse effects or ecological disturbances. In light of the existing links between the oral microbiome and systemic health, future studies should incorporate standardized safety outcomes and microbiome analyses to more accurately assess the biological implications of repeated mouthwash use.

Increasing evidence links the oral microbiome to systemic health and disease susceptibility. Therefore, understanding how repeated exposure to antiseptic agents might alter microbial balance is required. The absence of such data represents a significant gap that should be addressed in future investigations.

The evidence suggests that antiseptic mouthwashes may offer temporary and complementary benefits in reducing viral burden in saliva, mainly demonstrated for SARS-CoV-2, particularly in settings involving close contact and aerosol generation, such as dental and medical procedures [30,31,32]. From a clinical and dental perspective, their pre-procedural use can transiently lower the immediate risk of viral dissemination, providing a simple and inexpensive adjunct to standard infection-control protocols. This may be especially relevant in dentistry, where close patient proximity and exposure to oral fluids are unavoidable. Nevertheless, the effect is short-lived and mouthwashes should be regarded solely as short-term adjuncts, not as substitutes for personal protective equipment, vaccination, or other established preventive measures.

The efficacy of these measures is demonstrated by their ability to temporarily reduce viral presence in the oral cavity, which may consequently decrease short-term transmission risk. Nevertheless, it should be noted that these interventions do not appear to have any significant impact on disease progression or outcomes.

Antiseptic rinses act broadly, affecting not only viruses but also bacterial and fungal communities. While this non-specificity may reduce the overall microbial burden and lower infection risk, it also carries the potential to disrupt the ecological balance of the oral microbiome [36,37]. Repeated or long-term use could promote dysbiosis, altering protective bacterial populations and increasing susceptibility to opportunistic pathogens. This dimension requires further investigation in future trials.

Thus, mouthwashes represent a low-cost, widely accessible, and acceptable intervention. Their ease of use makes mouthwashes attractive as a rapid-response measure in situations where SARS-CoV-2 or other pathogens with documented oral presence may pose an acute transmission risk. However, the transient nature of their efficacy highlights the risk of a false sense of security if they are promoted without clear communication about their limitations.

Future studies should aim to address the gaps identified in this review by expanding investigations beyond SARS-CoV-2 to include other respiratory and oral viruses; adopting standardized methodologies for sampling, timing, and outcome assessment; Including longer follow-up periods to determine the persistence of antiviral effects; incorporating clinically meaningful endpoints, such as infectivity, transmission rate, and symptom evolution; evaluating safety profiles and microbiome alterations associated with repeated mouthwash use.

This scoping review has several limitations, even if it was conducted following the PRISMA-ScR process. First, the three-database search strategy attempted to obtain an accurate overview but may not have identified all available sources, especially those in the grey literature that were not included in the data search. The available evidence was also characterized by substantial methodological heterogeneity in study design, sample size, intervention protocols, and timing of viral load assessment, which prevented any quantitative synthesis or meta-analysis. All the included studies focused on SARS-CoV-2, limiting the extrapolation of conclusions to other viral infections with oral tropism. Furthermore, the majority of trials were short-term and relied on molecular surrogate markers rather than clinical or infectivity outcomes, reducing the strength of the evidence. Despite these limitations, this review provides a comprehensive synthesis of current evidence and highlights critical knowledge gaps that should guide the design of future standardized and longitudinal investigations.

## 5. Conclusions

This scoping review enabled the systematic mapping of the extant literature on saliva and antiseptic mouthwashes in the context of viral infections, thus identifying both the areas already explored and those that remain underinvestigated. The extant evidence on SARS-CoV-2 demonstrates that saliva represents a valuable diagnostic and surveillance medium and a potential vehicle for viral transmission, while antiseptic mouthwashes can transiently reduce salivary viral load. However, the majority of extant studies are limited to SARS-CoV-2, rely on heterogeneous methodologies, and provide only short-term observations without standardized clinical outcomes.

It is suggested that future research should build upon these findings by adopting harmonized study protocols, incorporating longer follow-up periods, and expanding the investigation to other respiratory and oral viruses. The establishment of consistent methodologies and clinically meaningful endpoints will be essential to clarify the role of saliva in viral pathogenesis and to define the realistic contribution of antiseptic mouthwashes as temporary, complementary measures within broader infection-control strategies. 

## Figures and Tables

**Table 1:** Mapping of the studies included in the scoping review. The table summarizes the characteristics of the included randomized controlled trials and the systematic review, detailing the study type, virus investigated, population, main research focus, and agents evaluated.

First Author (Year)	Study Type	Virus	Population	Theme Studied	Saliva Sampling Method	Intervention	Identified Gaps
Elzein (2021) [23]	RCT	SARS-CoV-2	COVID-19 patients	Mouthwashes	Self-collected cough-out saliva	CHX vs. PVP-I PVP-I	Short-term (≤2 h) effect; small sample size; lack of placebo group
Natto (2022) [17]	RCT	SARS-CoV-2	COVID-19 patients	Mouthwashes	Unstimulated saliva passive drool	PVP-I and H_2_O_2_	Transient effects; heterogeneous baseline viral load; no long-term assessment
Alsaleh (2024) [26]	RCT	SARS-CoV-2	COVID-19 patients	Mouthwashes	Oral/oropharyngeal swab	CHX, CPC, PVP-I	Modest viral load reduction; no follow-up beyond 2 h
Sulistyani (2024) [24]	RCT	SARS-CoV-2	Asymptomatic/mild COVID-19 patients	Mouthwashes	Oral/oropharyngeal swab	CHX, CPC, PVP-I	Limited sample; short follow-up
Alzahrani (2022) [18]	RCT	SARS-CoV-2	COVID-19 patients	Mouthwashes	Unstimulated saliva passive drool	CPC	Methodological heterogeneity
Perussolo (2023) [21]	RCT	SARS-CoV-2	Hospitalized COVID-19 patients	Mouthwashes	Unstimulated saliva, spitting	CPC	Mixed outcomes; underpowered; inconsistent endpoints
Onozuka (2024a) [20]	RCT	SARS-CoV-2	Mild COVID-19 patients	Mouthwashes	Unstimulated saliva, spitting	H_2_O_2_	Reduction only at 10 min; not sustained beyond 30–60 min
Onozuka (2024b) [34]	RCT	SARS-CoV-2	Asymptomatic/mild COVID-19	Mouthwashes	Oral/oropharyngeal swab	CHX	No significant difference vs. placebo; mild disease cases
Ferrer (2021) [16]	RCT	SARS-CoV-2	COVID-19 patients	Mouthwashes	Unstimulated saliva passive drool	CHX, H_2_O_2_, PVP-I	Ineffective in vivo; minimal reduction; no control of confounders
Costa (2022) [22]	RCT	SARS-CoV-2	COVID-19 patients	Mouthwashes	Unstimulated saliva, spitting	APD	Small sample; transient reduction; absence of control arm
Fantozzi (2022) [27]	RCT	SARS-CoV-2	COVID-19 patients	Mouthwashes	Oral/oropharyngeal swab	BAC	Pilot trial; limited generalizability
da Silva Santos (2021) [33]	RCT	SARS-CoV-2	Hospitalized COVID-19 patients	Mouthwashes	Oral/oropharyngeal swab	Hexetidine, Thymol, H_2_O_2_	Single-center; small cohort; not replicated
Meister (2022) [28]	RCT	SARS-CoV-2	COVID-19 patients	Mouthwashes	Oral/oropharyngeal swab	CDCM	Strong in vitro findings; weak in vivo evidence; transient outcome
Ogun (2022) [25]	RCT	SARS-CoV-2	COVID-19 patients	Mouthwashes	Oral/oropharyngeal swab	CHX, H_2_O_2_, PVP-I	Small pilot; no clinical outcome data; underpowered
Carrouel (2021) [29]	RCT	SARS-CoV-2	Asymptomatic/mild COVID-19 patients	Mouthwashes	Unstimulated saliva, standardized saliva collection kit	CHX vs. PVP-I	Limited follow-up (≤7 days); modest and variable effects
Shan (2025) [19]	RCT	SARS-CoV-2	COVID-19 patients pre-dental	Mouthwashes	Unstimulated saliva passive drool	PVP-I	Pre-procedural focus; transient effect; no virological endpoints
Lin (2023) [35]	SR	SARS-CoV-2	Multiple	Mouthwashes	Not applicable	PVP-I, CHX, CPC, H_2_O_2_, APD, BAC	Focused on SARS-CoV-2 only; heterogeneous study designs; variable quality
Verma (2021) [13]	SR	SARS-CoV-2	COVID-19 patients	Salivary diagnosis	Not applicable	-	Limited to SARS-CoV-2; no comparator; small and heterogeneous sample sizes
Zhang (2023) [14]	SR	SARS-CoV-2	COVID-19 patients	Salivary diagnosis	Not applicable	-	No long-term follow-up; inconsistent saliva collection methods
Espejo-Carrera (2025) [15]	SR	SARS-CoV-2	COVID-19 patients	Salivary diagnosis	Not applicable	-	Heterogeneous protocols; lack of longitudinal data;
Koletsi (2020) [30]	SR	SARS-CoV-2	Dental procedures	Salivary transmission	Not applicable	-	No viral quantification; heterogeneous aerosol measures; lack of clinical correlation
Garcìa-Sanchez (2022) [31]	SR	SARS-CoV-2	Dental procedures	Salivary transmission	Not applicable	PVP-I	No standardized aerosol quantification; small number of trials; focus on COVID-19 only
Ebrahimi 2023 [32]	SR	SARS-CoV-2	Dental procedures	Salivary transmission	Not applicable	PVP-I, CHX	Short-term outcomes; limited to SARS-CoV-2; no viral viability data

RCT, randomized clinical trial; SR, systematic review; CHX, chlorhexidine; PVP-I, povidone-iodine; CPC, cetylpyridinium chloride; H_2_O_2_, hydrogen peroxide; APD, Anionic phthalocyanine derivative; BAC, benzalkonium chloride; CDCM, β-cyclodextrin–citrox.

**Table 2:** Risk of bias assessment for systematic reviews and meta-analyses using the NIH quality assessment tool. In the color-coded ranking, green color represents low risk of bias, orange some concerns, and red high risk of bias.

First Author, Year	Focused Question	Eligibility Criteria	Literature Search Strategy	Independent Review	Independent Rate	Characteristics and Results	Bias Assessed	Heterogeneity Assessed
Lin et al., 2023 [35]								
Espejo-Carrera et al., 2025 [15]								
Verma et al., 2021 [13]								
Ebrahimi et al., 2023 [32]								
Koletsi et al., 2020 [30]								
Zhang et al., 2023 [14]								
Garcia-Sanchez et al., 2022 [31]								

**Table 3:** Risk of bias assessment for controlled intervention studies using the NIH quality assessment tool. In the color-coded ranking, green color represents low risk of bias, orange some concerns, and red high risk of bias.

First Author, Year	Type of Study	Method of Randomization	Treatment Allocation Concealed	Blinded Information	Blind Evaluation	Similar Group at Baseline	Overall Drop-out Rate	Differential Drop-out Rate	Adherence to the Intervention	Other Interventions	Outcomes Assessment	Sample Size	Outcomes Reported/Subgroups	Analyze of Randomized
da Silva Santos et al., 2021 [33]														
Ferrer et al., 2021 [16]														
Meister et al., 2022 [28]														
Elzein et al., 2021 [23]														
Natto et al., 2022 [17]														
Sulistyani et al., 2024 [24]														
Ogun et al., 2022 [25]														
Onozuka et al., 2024a [34]														
Perussolo et al., 2023 [21]														
Onozuka et al., 2024b [20]														
Alsaleh et al., 2024 [26]														
Alzahrani et al., 2022 [18]														
Costa et al., 2022 [22]														
Shan et al., 2025 [19]														
Fantozzi et al., 2022 [27]														
Carrouel et al., 2021 [29]														

## Data Availability

No new data were created or analyzed in this study. Data sharing is not applicable to this article.

## Supplementary Materials

The following supporting information can be downloaded at: https://www.mdpi.com/article/10.3390/v17111509/s1, Table S1: Preferred Reporting Items for Systematic reviews and Meta-Analyses extension for Scoping Reviews (PRISMA-ScR) Checklist.
